# FRAILTY TRAJECTORIES IN BRAZILIAN COMMUNITY-DWELLING OLDER ADULTS: A 9-YEAR FOLLOW-UP STUDY

**DOI:** 10.1093/geroni/igae098.3985

**Published:** 2024-12-31

**Authors:** Ruth Melo, Daniela Assumpção, Meire Cachioni, Deusivania Falcão, Samila Sathler Tavares Batistoni, Flávia Silva Arbex Borim, Anita Liberalesso Neri, Mônica Sanches Yassuda

**Affiliations:** University of São Paulo, São Paulo, Sao Paulo, Brazil; Universidade Estadual de Campinas (UNICAMP), Campinas, Sao Paulo, Brazil; University São Paulo, São Paulo, Sao Paulo, Brazil; USP - University of Sao Paulo, USP - University of Sao Paulo, Sao Paulo, Brazil; University of São Paulo, University of São Paulo, Sao Paulo, Brazil; Universidade Estadual de Campinas, Universidade Estadual de Campinas, Sao Paulo, Brazil; State University of Campinas, State University of Campinas, Sao Paulo, Brazil; University of São Paulo, University of São Paulo, Sao Paulo, Brazil

## Abstract

This prospective observational study analyzed the factors influencing frailty trajectories among Brazilian community-dwelling older adults over a nine-year follow-up period. Sociodemographic data, self-assessed health, multimorbidities, social support, advanced daily living activities (AADL), cognition, and frailty phenotype were selected for analysis from the Fibra (Frailty in Brazilian Elderly) study database. Incidence was calculated based on new cases (pre-frailty/frailty) at follow-up versus phenotype (robust/pre-frailty) at baseline. Pearson’s chi-square test was used for intergroup comparison (i.e., progression status). The relative risk (RR) for frailty progression and reversal was calculated using logistic regression, both in crude (c) and adjusted (a) forms. After nine years, the proportion of older adults classified as frail increased (11.9% to 18.1%), while the proportion of those classified as robust decreased (30.1% to 13.8%). The incidence of frailty and pre-frailty was 22.9% and 49.4%, respectively. Advanced age (RRa:3.41; p=0.001), cognitive impairment (RRa:2.06, p=0.002), illiteracy (RRa:1.70; p=0.012), male sex (RRa:1.68; p=0.044) and non-performance/abandonment of AADL (RRa:1.48; p=0.007) were predictors of worsening/death in follow-up. Only living alone was protective against frailty. After nine years, there was an increase in older adults living with frailty and a reduction in the ones considered robust, with an incidence of frailty of 22.9%. Different factors predicted worsening/death after nine years. Future longitudinal studies should improve the understanding of risk factors for frailty progression.

